# Supplementary material for: Accurate and fast identification of minimally prepared bacteria phenotypes using Raman spectroscopy assisted by machine learning
Source: Sci Rep. 2022 Sep 30;12:16436. doi: 10.1038/s41598-022-20850-z (PMC9524333; doi:10.1038/s41598-022-20850-z)
Supplement: Supplementary file 1 — Supplementary Information. [file 41598_2022_20850_MOESM1_ESM.pdf]

SUPPLEMENTARY MATERIAL

Identification of Escherichia coli isolates and antibiotic resistance profiles

The Escherichia coli isolates D4282 (P1), D4310 (P2), and D4211 (P3) were isolated from urine and were species identified from indole spot test (positive) and from plating on CHROMID® CPS ELITE agar plates (Biomérieux, USA). Resistance patterns for the clinical isolates and for the ATCC strains were identified at the Odense University Hospital Clinical Microbiology Laboratory by measuring inhibition zone diameter using the disk diffusion test.

	P1 (D4282)	P2 (D4310)	P3 (D4211)	ATCC25922	ATCC35218
Amoxycillin/Clavulanic acid	+++	-	-	+++	+++
Ampicillin	+++	-	-	+++	-
Ciprofloxacin	+++	+++	+++	+++	+++
Cefpodoxim	+++	+++	+++	+++	+++
Mecillinam	+++	-	+++	+++	+++
Meropenem	+++	+++	+++	+++	+++
Gentamicin	+++	+++	+++	+++	+++
Cefuroxime	+++	+	+++	+++	+++
Nitrofurantoin	+++	+++	+++	+++	+++
Sulfanomide	+++	+++	-	+++	-
Piperacillin/tazobactam	+++	-	+++	+++	+++
Trimethroprim	+++	+++	+++	+++	+++

FIG. 1. Resistance profiles for E. coli isolates and E. coli ATCC strains.

Computation-time comparison

Training and inference times of CNN and ST models.

**Model**	**Training, inference (seconds)**			
	**Setup 1**		**Setup 2**	
	E. coli binary BZ = 10	E. coli binary BZ =100	E. coli binary BZ = 10	E. coli binary BZ = 100
CNN	1884.4, 60.6	1708.5, 52.9	163.9, 6.5	83.9, 3.5
ST(1,2,7)	229.9, 3.5	44, 1.2	45, 2.1	7.8, 0.42
ST-pe(1,10,3)	143.5, 2.6	27.9, 0.8	46.2, 2.1	7.8, 0.41

Setup 1: Intel(R) Xeon(R) @ 2.20GHz CPU.
Setup 2: Tesla K80 GPU and Intel(R)Xeon(R) @ 2.30GHz CPU.

FIG. 2. For the tests of computation time, the code was optimized individually for each of the two setups and the results are the sum of 10 epochs. The ST took substantially shorter time to compute than the CNN. It should however be noted that a small amount of the difference may be due to differences in hyperparameters, such as weight decay, parameter amount, and learning rate, and that the difference therefore cannot solely be attributed to the model architectures.

Figure 2 shows a comparison in compute time between the CNN benchmark model and two different ST architectures developed and used in this work. We generally observe that the ST models are significantly faster to train and to use for inference. We attribute this performance difference to a low ST model depth, which simultaneously helps to avoid model overfitting.

Pseudocode for the NoiseMix algorithm

```
PSEUDOCODE FOR NOISEMIX:
FOR epoch in range epochs:
    X,y = List, List
    FOR class  data, label in (training  data, labels):    # Class balancing
        shuffle class  data
        extend X with amount of class  data
        extend y with amount entries of label
    data = List
    FOR datapoint, label in (X,y):                                    # Start of noise mixing.
        FOR mixing  class  data, mixing  class  label, weight  range  in (mixing  data, mixing  labels, weight  ranges):
            IF NOT (label == background  label AND  mixing  class  label != background  label):
                broadcast to datapoint, a random spectra from (mixing  class  data multiplied by a random number from weight  range)
        subtract from datapoint, the linear function between the start and end values of datapoint     # End of noise mixing.
        normalise datapoint between of 0-1

        append datapoint to data
    dataset = CALL create  dataset(data, shuffle=True)
Train model with dataset:
```

FIG. 3. Pseudocode for the `NoiseMix` algorithm.
